# *Adhatoda Vasica* attenuates inflammatory and hypoxic responses in preclinical mouse models: potential for repurposing in COVID-19-like conditions

**DOI:** 10.1186/s12931-021-01698-9

**Published:** 2021-04-06

**Authors:** Atish Gheware, Dhwani Dholakia, Sadasivam Kannan, Lipsa Panda, Ritu Rani, Bijay Ranjan Pattnaik, Vaibhav Jain, Yash Parekh, M. Ghalib Enayathullah, Kiran Kumar Bokara, Venkatesan Subramanian, Mitali Mukerji, Anurag Agrawal, Bhavana Prasher

**Affiliations:** 1 Genomics and Molecular Medicine, Council of Scientific and Industrial Research -Institute of Genomics and Integrative Biology (CSIR-IGIB), Delhi, 110007 India; 2 CSIR’s Ayurgenomics Unit–TRISUTRA (Translational Research and Innovative Science ThRoughAyurgenomics) CSIR-IGIB, Delhi, 110007 India; 3 Centre of Excellence for Applied Development of Ayurveda, Prakriti and Genomics, CSIR’s Ayurgenomics Unit–TRISUTRA (Translational Research and Innovative Science ThRoughAyurgenomics), CSIR- IGIB, Delhi, 110007 India; 4 Center for Translational Research in Lung Disease, CSIR- IGIB, Delhi, 110007 India; 5 Center for High Computing, CSIR- Central Leather Research Institute (CLRI), Chennai, 600020 India; 6 CSIR-Center for Cellular and Molecular Biology, Annexe-II, Medical Biotechnology Complex, Uppal Road, Hyderabad, Telangana 500007 India; 7 Academy of Scientific and Innovative Research (AcSIR), Ghaziabad, 201002 India

**Keywords:** Hypoxia, Fibrosis, Sepsis, Angiogenesis, Blood coagulation, SARS-CoV2, COVID-19, *Adhatoda Vasica*

## Abstract

**Background:**

COVID-19 pneumonia has been associated with severe acute hypoxia, sepsis-like states, thrombosis and chronic sequelae including persisting hypoxia and fibrosis. The molecular hypoxia response pathway has been associated with such pathologies and our recent observations on anti-hypoxic and anti-inflammatory effects of whole aqueous extract *of Adhatoda Vasica* (AV) prompted us to explore its effects on relevant preclinical mouse models.

**Methods:**

In this study, we tested the effect of whole aqueous extract of AV, in murine models of bleomycin induced pulmonary fibrosis, Cecum Ligation and Puncture (CLP) induced sepsis, and siRNA induced hypoxia-thrombosis phenotype. The effect on lung of AV treated naïve mice was also studied at transcriptome level. We also determined if the extract may have any effect on SARS-CoV2 replication.

**Results:**

Oral administration AV extract attenuates increased airway inflammation, levels of transforming growth factor-β1 (TGF-β1), IL-6, HIF-1α and improves the overall survival rates of mice in the models of pulmonary fibrosis and sepsis and rescues the siRNA induced inflammation and associated blood coagulation phenotypes in mice. We observed downregulation of hypoxia, inflammation, TGF-β1, and angiogenesis genes and upregulation of adaptive immunity-related genes in the lung transcriptome. AV treatment also reduced the viral load in Vero cells infected with SARS-CoV2.

**Conclusion:**

Our results provide a scientific rationale for this ayurvedic herbal medicine in ameliorating the hypoxia-hyperinflammation features and highlights the repurposing potential of AV in COVID-19-like conditions.

**Supplementary Information:**

The online version contains supplementary material available at 10.1186/s12931-021-01698-9.

## Background

Increased alveolar hypoxic response levels are inevitable consequences of many respiratory disorders such as chronic obstructive pulmonary disease and pulmonary fibrosis [1, 2]. The key player of cellular response to hypoxia is the hypoxia-inducible factor (HIF)-1α and its regulatory protein, the prolyl hydroxylase domain (PHD)-2 enzyme [3]. The induction of HIF-1α is considered to be pro-inflammatory. It leads to transcriptional activation of essential genes implicated in airway remodelling and inflammation, such as vascular endothelial growth factor, transforming growth factor-beta 1 (TGF-β1), inducible nitric oxide synthase, interleukin -17 (IL-17), and IL-6 [3, 4]. Thus, it is not just a consequence of diseases, elevated tissue/cellular hypoxia actively participates in exaggerating the inflammatory response contributing to progressive lung damage/injury.

HIF-1α also plays a pivotal role in infection, especially in promoting viral and bacterial replication [5]. In the present COVID-19 pandemic caused by the severe acute respiratory coronavirus 2 (SARS-CoV2), the role of hypoxia response in inducing severe lung inflammation and other outcomes has been one of the most highlighted observations [6–10]. Clinically, the interaction of the host and SARS-CoV2 is broadly described in three stages: first, asymptomatic state; second, a non-severe symptomatic state characterized by upper airway and conducting airway response; third, severe respiratory symptomatic state with the presence of hypoxia, acute respiratory distress syndrome (ARDS) and progression to sepsis [7]. During incubation and non-severe state, a specific humoral and cell-mediated adaptive immune response is required to eradicate the virus and prevent disease progression to a severe condition. Thus, strategies to boost immune responses at this stage are undoubtedly important [7, 11]. However, defective immune response causes further accumulation of immune cells in the lungs, progressing to aggressive production of a pro-inflammatory cytokine such as IL-6, TNF-α resulting in an influx of immune cells and cytokines that damage the airways/ lung architecture. This extended release of cytokines by the immune system in response to the viral infection and/or secondary infections causes severe inflammation, endothelial dysfunction, sepsis and multi-organ damage [7, 11, 12]. In addition, recent research also reports coagulation abnormalities in severe COVID-19 cases [13]. The relation of hypoxia-coagulation is well known, where we and others also showed the crucial role of cellular hypoxic response in the form of thrombosis and bleeding susceptibility through HIF-1α and vWF axis [14, 15]. Thus, medicinal agents that possess immune-boosting and anti-hypoxic effects could hold a promise for a better therapeutic option to preclude the SARS-CoV2 infection and severity.

We have recently shown an extract of *Adhatoda Vasica* (AV); an ayurvedic medicine that possesses robust anti-hypoxic properties and can reduce severe airway inflammation induced by an augmented hypoxic response in treatment-resistant asthmatic mice [16]. The anti-HIF-1α effect of AV also restores the cellular hypoxia-mediated loss of mitochondrial morphofunction in vitro [16]. As a follow-up, we evaluated AV’s usefulness in other severe lung pathologies, where hypoxia signalling is pertinent, and which are relevant to the clinical course of COVID-19 namely lung injury, fibrosis, and thrombosis. Since viral proliferation may be altered by molecular modulation of such pathways, we further tested the potential of AV in limiting SARS-CoV2 proliferation.

## Methods

### Preparation of plant extract and LC–MS fingerprinting

*Adhatoda Vasica* (AV) was collected from Delhi-NCR region, India in the flowering season (November to March). Water extract of plant (leaves, twigs and flowers) was prepared according to classical method described for rasakriya in *Caraka Samhita* [17]. The process for the formulation involved preparation of decoction condensation and drying as described in earlier study [18]. Chemical fingerprinting of prepared AV extract was carried out by LC–MS at SAIF, CSIR-CDRI, Lucknow, India.

### Animals

The study was designed and performed following guidelines of the Committee for the Purpose of Control and Supervision of Experiments on Animals (CPCSEA) and approved by Institutional Animal Ethics Committee of CSIR-Institute of Genomics & Integrative Biology (IGIB), New Delhi, India. The BALB/c and C57BL/6 male mice (8–10 weeks old) were bred under the pathogen-free condition. They were acclimatized to animal house environment one week before starting the experiments at CSIR-IGIB, New Delhi, India and maintained according to guidelines of CPCSEA. All the surgical procedures were performed under sodium pentobarbital anaesthesia and maximum efforts are taken for minimum suffering of animals.

### Grouping and treatment of mice

**Fig. 1:**
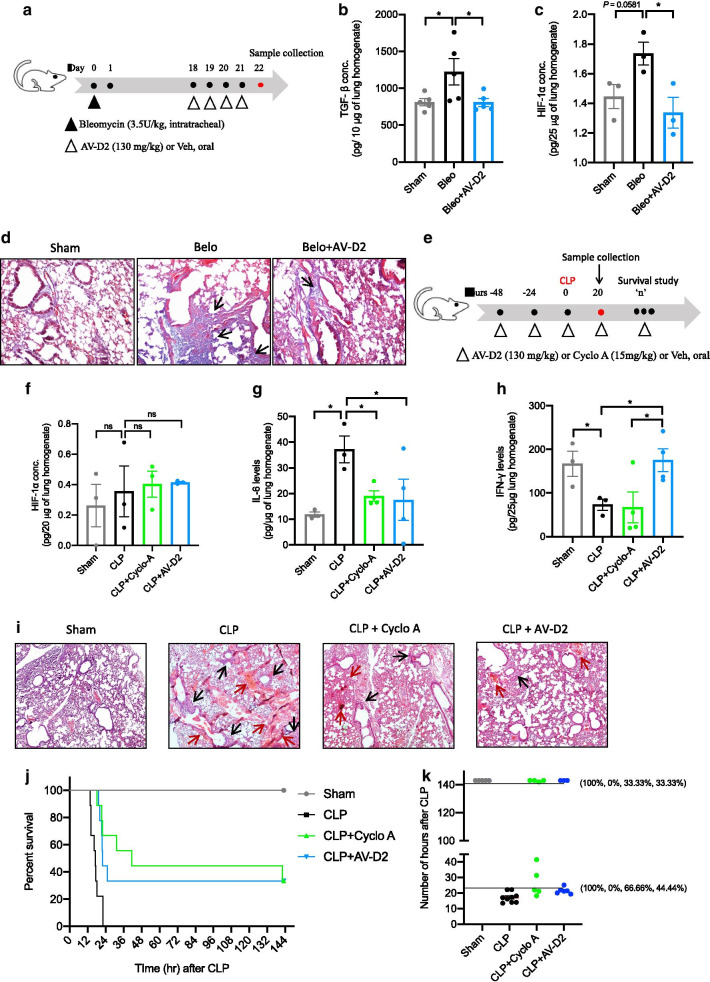
AV reduces the bleomycin and CLP induced inflammatory features of mice lungs. **a** Schematic representation of bleomycin-induced pulmonary fibrosis model of mice. Male C57BL/6 mice were treated with bleomycin on day 0 by the intratracheal route. After genesis of fibrosis features, Bleo mice therapeutically treated with AV-D2 dose (130mg/kg) from day 18–21. On day 22, mice sacrificed to collect the sample as described in methods.  ELISA for** b** TGF-β1 **c** and HIF-1α levels in mice lung homogenate. **d **Representative photomicrographs of fixed mouse lung tissue sections stained with Masson’s Trichome (MT) (10× magnification). Positive staining indicated by black arrow. **e** Schematic representation of Cecum ligation and puncture (CLP) mice model protocol. Two days before the CLP surgery, Cyclo A (15 mg/kg) and AV-D2 (130 mg/kg) treatment was started, on day 0 CLP was done and after 4 hours of surgery Cyclo A (15 mg/kg) and AV-D2 (130 mg/kg) treatment was done and continued till mice survive (‘n’ hours). To assess cytokines levels and lung histology after CLP,  3-4 mice from each group was sacrificed after 20 hours of CLP as described in methods. **f **HIF-1α **g** IL-6 **h** and IFN-g levels in mice lung homogenate after 20 hours of surgery. **i** Representative photomicrographs of fixed mouse lung tissue sections stained with H&E (4× magnification) to asses lung inflammation (indicated by black arrow) and associated blood exudation (indicated by red arrow). **j** Survival curve study for 142 hours in CLP induced sepsis mouse. **k** Number of hour’s survival plotted for each group showing % survival after 24 and 142 hours in bracket (n= 5-9).Data are shown as mean ±SEM of three to five mice per group. Significance denoted by *P≤0.05, **P≤0.01, ***P≤0.001 and ****P≤0.0001 and determined  by  ordinary one-way ANOVA) using GraphPad. ns represents the non-significant. Significance of survival curve was determined by log-rank (Mantel-Cox) test using GraphPad

**Fig. 2:**
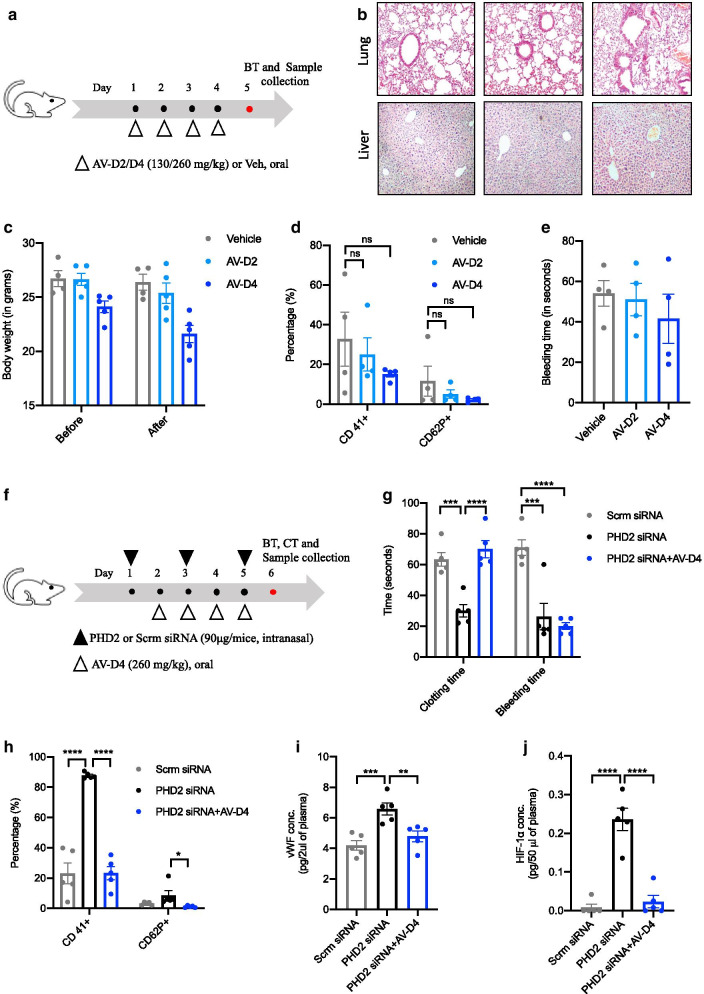
AV treatment to mice is protective against the blood coagulation phenotype in HIF-1α dependent manner. **a** Schematic representation of AV treatment protocol to naïve BALB/c mice. AV treatment was given for 4 consecutive days using AV-D2 (130 mg/kg) and AV-D4 dose (260 mg/kg). **b **Representative photomicrographs of fixed mouse lung and liver tissue sections stained with H&E (4× magnification) to asses AV mediated any effect on histological architecture in terms of inflammation. **c** Mice body weights measured before the start of the AV or Veh treatment and after the completion of treatment (AV or Veh) on day 5th of the protocol. **d** Total (CD41+) and active platelet (CD 62P+) count in mice whole blood (anticoagulated) measured by flow cytometry. **e** Tail bleeding time (in seconds) measured in Vehicle or AV treated mice as per the method described in the protocol. **f** Schematic representation of the siRNA experiment protocol. Scrambled or PHD2 siRNA was given intranasally to mice on days 1, 3, and 5. AV-D4 treatment was started therapeutically from day second and continued till 5h day of protocol as described in methods. On day six, mice were subjected to tail bleeding and clotting time assay. **g** Clotting and tail bleeding time (in seconds) measured in mice groups as described in methods. **h **Total (CD41+) and active platelet (CD 62P+) count assessed in mouse whole blood via flow cytometry. ELISA for estimation of **i** vWF and **j** HIF-1α levels in mouse plasma. Data are shown as mean ±SEM of four to five mice per group. Term ns represents the non-significant. Significance denoted by *P≤0.05, **P≤0.01, ***P≤0.001 and ****P≤0.0001 and determined by  ordinary one way ANOVA) using GraphPad

Mice were mainly divided into two groups as Vehicle and treatment according to the experiment. In the case of Cecum ligation puncture (CLP) survival study experiment, BALB/c mice were divided into Sham (Control mice, distil water and 10% ethanol, oral, *n* = 5) and CLP (mice underwent CLP surgery, *n* = 9). CLP mice subdivide in CLP + Cyclo A (Cyclosporin A treated CLP mice, *n* = 9) and CLP + AV-D2 (*Adhatoda Vasica* extract-treated CLP mice, *n* = 9) group. Treatment of AV (130 mg/kg dissolved in distilled water, oral, two times a day) or Cyclo A (Cyclosporin A, 15 mg/kg dissolved in 10% ethanol, oral, once a day) was started two days (48hours) before CLP and was continued till the mice survive after surgery (Fig. 1). To assess lung histology and cytokine levels in the CLP experiment, mice sacrificed after 20 h of surgery from Sham (*n* = 3), CLP alone (*n* = 3), CLP + Cyclo A (*n* = 4), and CLP + AV-D2 (*n* = 4) group. Similarly, in the bleomycin fibrosis model (*n* = 5 mice/group), C57BL/6 mice were divided into Vehicle (i.e., Sham), Bleo (bleomycin treated), and Bleo + AV-D2 (AV 130 mg/kg treated Bleo mice, oral, two times a day). In that, AV treatment was done from day 18 to 21, as shown in the schematic (Fig. 1a). Bleomycin (3.5 U/kg of mice) was given intratracheally to isoflurane-anesthetised C57BL/6 mice on day 0 of the protocol (Fig. 1) to induce fibrotic changes in mice as described previously [19]. For transcriptomic study, BALB/c mice were divided into Vehicle (distil water, oral, two times a day, *n* = 4) and *Adhatoda Vasica* (AV) extract group. AV group was further subdivided according to its dose: AV-D2 (*Adhatoda Vasica* extract 130 mg/kg, dissolved in distilled water, oral, two times a day, *n* = 5) and AV-D4 (*Adhatoda Vasica* extract 260 mg/kg, dissolved in distilled water, oral, two times a day, *n* = 5) as described previously [16]. Distil water or AV (130 mg/kg or 260 mg/kg) treatment was given to mice by oral gavage for four consecutive days, as represented in the Fig. 2a. In PHD2 siRNA-induced hypoxia model (*n* = 5 mice/group), BALB/c mice were divided into scrambled siRNA (Scrm siRNA), prolyl hydroxylase domain-2 siRNA (PHD2 siRNA), and AV-D4 treated PHD2 siRNA group (PHD2 siRNA + AV-D4) group. AV-D4 dose (260 mg/kg, dissolved in distilled water, oral, two times a day) given for four consecutive days and 90 µg siRNA (Sigma) administered intranasally which dissolved in ultrapure DNase and RNAse free water with in-vivo jetPEI as the transfection reagent (Polyplus Transfection, France) to isoflurane-anesthetised mice on day 1, 3 and 5th of the protocol.

### Clotting and bleeding time assay, blood collection and platelet measurement

Tail bleeding was measured as described previously [15]. Briefly, anesthetized mice's tail amputated with a sharp scalpel, and bleeding time was then determined by monitoring the duration of animal tail bleeding until it ceased and was kept in a prone position and immersed in PBS. Clotting time measured by the capillary tube method. The mice's tail was cleaned with 70% alcohol and punctured with a 1 ml syringe needle. Filled two capillary tubes with free-flowing blood from the puncture site after wiping the first drop of blood. Stop clock started and capillary tubes were broken to see whether a thin fibrin stand formed between two broken ends. After fibrin stand is observed, clotting time measured from the average of two capillary tubes. For platelet measurement, blood obtained by cardiac puncture and collected in EDTA coated MiniCollect tubes (Greiner Bio-One Gmbh, kremsmünster, Austria) as described [15]. The whole blood was used to measure total and of active Platelet count and was carried out through flow cytometry using FACSCalibur (BD Biosciences, USA). Briefly, diluted whole blood (1:4) in PBS was incubated with APC conjugated anti-CD62P (eBioscience Inc, San Diego, CA, USA) and FITC conjugated anti-CD41 (eBioscience Inc, San Diego, CA, USA) for 15 min. Matched fluorescein-conjugated isotype control antibodies were used simultaneously for staining for comparison. The activity was compared using CellQuest Pro software (BD Biosciences, USA).

### Bronchoalveolar lavage fluid collection and histopathology

Bronchoalveolar lavage fluid (BAL) was collected by instilling 1 ml PBS into the tracheotomised airway and recovered BAL fluids were processed to get total leukocyte count, as described previously [16, 20]. For lung histology, the lungs were excised and fixed in 10% buffered formalin. The fixed, paraffin-embedded tissues cut into 5um sections and either stained with hematoxylin and eosin (H&E) to asses inflammation or Masson’s trichome (MT) staining to assess collagen content.

### Cecum ligation puncture (CLP) procedure

Mice were anesthetised by injecting intraperitoneally a solution of 1:1 ketamine (75 mg/kg) and xylazine (15 mg/kg). The abdomen was shaved, and the peritoneum area was disinfected betadine solution followed by wiping with a 70% alcohol. Under aseptic conditions, a 1 cm midline incision was made, and the cecum carefully exposed with the adjoining intestine. The cecum was then tightly ligated with a 3.0 Mersilk (PROLENE, 8680G; Ethicon) sutures at the base and punctured once with a 19-gauge needle on the same side of the cecum. A small amount of stool extruded to ensure patency of the puncture sites. The cecum then returned to the peritoneal cavity, and the wound was closed with 3.0 Mersilk sutures. Control mice (i.e. Sham), the cecum was exposed out and then returned to the peritoneum without ligation or puncture. Mice were resuscitated by injecting subcutaneously 1 ml of pre-warmed 0.9% saline solution using a 25G needle. After surgery, animals placed immediately to a cage with exposure to a heating lamp of 150 W until they recovered from the anaesthesia. The recovery time is from 30 min to 1 h. Mice were monitored every 12 h for survival or euthanised after 20 h (*n* = 3–4) for measurement of cytokines while they were fed with their regular diet and water. Two independent experiments recorded the mortality of mice after CLP surgery.

### TGF-b, IL-6,IFN-γ, HIF-1α, PHD2, and vWF measurement

The levels of TGF-b, IL-6, IFN-γ (BD, USA), HIF-1α (R&D, USA), PHD2 ((USCN, China) and vWF (USCN, China) were measured in lung tissue homogenate or in plasma of the mice by sandwich ELISA, as per manufacturer’s protocol.

### RNA isolation and whole transcriptome analysis

Total RNA was isolated from mouse lung tissue treated with AV (AVD2 and AV-D4) or distilled water (vehicle) using the RNeasy Plus Mini Kit (Qiagen, CA, USA) following the manufacturer's protocol. For genome-wide expression analysis, the Affymetrix GeneChip MTA 1.0 array was used according to the manufacturer's instruction. For each sample, 250 ng of RNA was quantified and hybridized to microarray chips following a series of consecutive steps described in the protocol. After hybridization, microarray chips are then scanned using an Affymetrix GCS 3,000 scanner (Affymetrix, CA, USA) and the signal values are further evaluated using the Affymetrix® GeneChip™ Command Console software. Raw data automatically extracted using the Affymetrix data extraction protocol in the Affymetrix GeneChip® Command Console® Software (AGCC). CEL file import, mRNA level, all analysis, and export of the results were all performed using Affymetrix® Expression Console™ software. A comparative study between the vehicle and the AV treated samples done. Genes considered to the differentially expressed by applying the criteria of significance p-value less than or equal to 0.05.

### Functional enrichment and Connectivity map analysis

For functional analysis, we used Enrichr (amp.pharm.mssm.edu) tool. For pathway and gene ontology analysis, we examined gene enrichment in Cellular Compartment, Biological Processes, BioPlanet, Wiki, KEGG human pathway and gene set enrichment was considered if P-value less than 0.05 in Enrich r tool. For connectivity map (CMap) analysis, differentially expressed genes ranked according to fold change and list of top 150 up and down-regulated genes compatible with the CMAP data signatures was used to query the connectivity using clue.io touchstone database. A positive score in CMap analysis indicates a similar expression pattern of AV with compared compounds’ gene expression signature, whereas a negative score indicates an opposite pattern.

### Molecular docking

The complete genome sequence of the novel SARS-CoV-2 virus was obtained from the National Centre for Biotechnology Information (NCBI) nucleotide database (NC_045512.2). The available 3D crystal structures of all the target proteins such as 3CLpro, PLpro, RdRp, S-protein, ACE2 and JAK2 were taken from protein data bank [21]. Others structures (NSP4, NSP7, NSP8, NSP9, NSP13, NSP14, NSP15 and NSP16, and TMPRSS2) were built using homology modelling with suitable templates using Swiss model [22] and I-TASEER web-servers [23]. The active regions of the proteins were identified by COACH meta-server and the results were compared with results from CASTp web server [24]. The impact on SARS-COV2 target protein were investigated for the compounds of *Adhatoda Vasica* as well as known antiviral, antimalarial and JAK inhibitors compounds by Molecular Docking studies using Schrodinger suite (Maestro) [25] and AutoDock vina packages [26]. In Schrodinger suite, all the target proteins were prepared using protein preparation wizard that included optimization followed by minimization of heavy atoms of proteins. The energy minimized 3D structures of all the ligands were prepared using LigPrep. The best pose of ligands that fit well in the protein cavity was carried out using OPLS3 force field with Glide package in Extra Precision mode (XP) mode. According to the size of binding cavity of the proteins, the coordinates x, y and z of the grid box were chosen with the grid resolution of 1 Å for calculations using AutoDock vina package.

### Cellular model, drug treatment and detection of SARS-CoV-2 using a qPCR assay

Vero cells were maintained in Dulbeco Minimum Essential Medium (Gibco) containing 10% Fetal Bovine Serum (Gibco) at 37 °C, 5% CO_2_. Cells were seeded into 96-well tissue culture plates 24 h prior to infection with SARS-CoV2 (Indian/a3i clade/2020 isolate) in BSL3 lab of CSIR-CCMB. The effect of (AV) aqueous extract was tested against the SARS-CoV2 virus [27] by taking different concentrations of AV: 100, 50, 12.5, 6.25 (µg/mL) in DMEM media. Briefly, the cells were primed with the AV for 2 h. The virus inoculum (0.1 MOI) was added to the cells along with different concentrations of AV and were left for infection for 3 h. Post-infection, viral inoculum was replaced with fresh media containing 10% FBS and were maintained at 37 °C, 5% CO_2_ until 72 h. After 72 h, cell supernatant was collected and spun for 10 min at 6,000 g to remove debris and the supernatant was transferred to fresh collection tubes. RNA was extracted from 200 μL aliquots of sample supernatant using the MagMAX™ Viral/Pathogen Extraction Kit (Applied Biosystems, Thermofisher). Extraction of viral RNA was carried out according to the manufacturer’s instructions. The viral supernatants from the test groups were added into the deep well plate (KingFisher™ Thermo Scientific) along with a lysis buffer containing the following components—MagMAX™ Viral/Pathogen Binding Solution; MVP-II Binding Beads; MagMAX™ Viral /Pathogen Proteinase-K of 260 μL; 10 μL; 5 μL respectively for 200μL of sample. RNA extraction was performed using KingFisher Flex (version 1.01, Thermo Scientific) by following manufactures instructions. The eluted RNA was immediately stored in -80 °C until further use. The detection of SARS-CoV2 was done using COVID-19 RT-PCR Detection Kit (Fosun 2019-nCoV qPCR, Shanghai Fosun Long March Medical Science Co. Ltd.) according to the manufacturer’s instructions. The kit detects Envelope gene (E; ROX labelled), Nucleocapsid gene (N- JOE labelled) and open reading frame1ab (ORF1ab, FAM labelled) specific to SARS-CoV2 for detection and amplification of the cDNA. SARS-CoV-2 cDNA (Ct ~ 28) was used as a positive control. The log viral particles and a semi–log graph was plotted through the linear regression equation obtained using the RNA extracted from the known viral particles by RT-qPCR, using N- and ORF1ab genes specific to SARS CoV2 virus and percent viral reduction was calculated, as described previously [28]. However, Ct value of N gene is considered to calculate the % viral reduction [28].

### Statistical analysis

Statistical significance determined by one-way analysis of variance and analysis was done using GraphPad Prism software. In the case of mice experiment, all data represent mean ± SEM; n = 3–10 in each group and significance denoted by *p < 0.05, **p < 0.01, ***p < 0.001. *p*-value > 0.05 is considered non-significant (NS). Significance of the survival study determined by Log-rank (Mantel-Cox) test using GraphPad Prism software.

## Results

### AV treatments inhibits the bleomycin induced pulmonary fibrosis features as well as increased HIF-1α levels in mice

To test the effect of AV treatment on lung fibrosis, bleomycin treated mice were orally administered with AV (130 mg/kg, AV-D2) as shown in Fig. 1a. We observed a significant increase in TGF-β1 and HIF-1α levels in bleomycin (Bleo) treated mice lung than control-Sham mice, which decreased after AV-D2 treatment (Fig. 1b, c). Masson's trichrome staining showed a marked increase in collagen deposition in Bleo mice lungs compared to Sham mice (Fig. 1d). AV-D2 treatment reduces this increased collagen deposition in Bleo mice (Fig. 1d).

### AV ameliorates the hallmarks of lung inflammation and injury in mice model of sepsis

Mice that underwent CLP (Cecal ligation and puncture) surgery did not show significant increase in HIF-1α levels after 20 h of surgery (Fig. 1f). Still, its downstream target, such IL-6, was significantly increased (Fig. 1g), and IFN-γ was decreased (Fig. 1h) in lung homogenate after 20 h of surgery compared to sham mice. AV pre-treatment restored the levels of both cytokines in mice lungs, whereas Cyclo-A (a positive control) pre-treatment reduced only IL-6 levels in mice (Fig. 1g, h). Besides, histological analyses showed that CLP mice lung sections stained with haematoxylin and eosin (H&E) had increased inflammation and blood exudation (Fig. 1i, Additional file 1: Fig. S5a). Pre-treatment of AV-D2 and Cyclo A to CLP mice showed reduced inflammation and blood exudation in lung histological sections (Fig. 1I). CLP surgery also leads to a significant decrease in mice survival rate compared to sham group (Fig. 1j). Treatment of Cyclo-A or AV-D2 to CLP mice significantly increases their survival rate compared to CLP untreated mice (Fig. 1j). In CLP + Cyclo A and CLP + AV-D2 group, the mice survival rate after 24 h is 66.6 and 44.4%, respectively (Fig. 1k). Though, in both groups, survival rate is 33.33% at the end of 142 h of CLP surgery (Fig. 1k).

### AV treatment inhibits hemostatic outcomes of hypoxia response induced by PHD2 siRNA in mice

Next, to test whether anti- HIF-1α effects of AV also prevents the blood coagulation phenotype [15], we treated naïve BALB/c mice with AV-D2 (130 mg/kg) and AV-D4 (260 mg/kg) concentration (Fig. 2a). Oral administration of AV-D2 and AV-D4 to naïve mice does not cause any significant change in body weight and lung and liver histological architecture (Fig. 2b, c, and Additional file 1: Fig. S5b), indicating its non-toxic nature in the tested doses. In the hemostasis parameter, treatment of AV-D4 dose to naïve healthy BALB/c mice causes a decrease in total as well as activated platelet count, although not statistically significant. Still, it does not affect mice tail bleeding time (Fig. 2d, e). To confirm the above-observed effect of AV-D4 on blood parameters, we induce cellular hypoxia response in mice by specific PHD2 siRNA treatment (Fig. 2f), as described previously [15]. PHD2 siRNA treatment (Additional file 1: Fig. S1a) leads to a significant decrease in blood clotting and tail bleeding time (Fig. 2g). It also causes an overall increase in total and activated platelet count in mice blood (Fig. 2h). These changes induced by PHD2 siRNA are associated with increased blood HIF-1α and vWF levels, indicating the development of platelet aggregation (Fig. 2i, j). Interestingly, AV-D4 treatment to PHD2 siRNA mice causes a significant reversal of blood coagulation phenotype in terms of mice's blood clotting time, platelet count (total and active), and vWF levels (Fig. 2g–i). These effects of AV are associated with the reversal of increased blood HIF-1α levels (Fig. 2j). However, AV treatment does not affect the mice's bleeding time, which was reduced after PHD2 siRNA treatment (Fig. 2g).

### Modulation of immune response and hypoxia pathway genes: revealed from lung transcriptome of AV treated mice

**Fig. 3:**
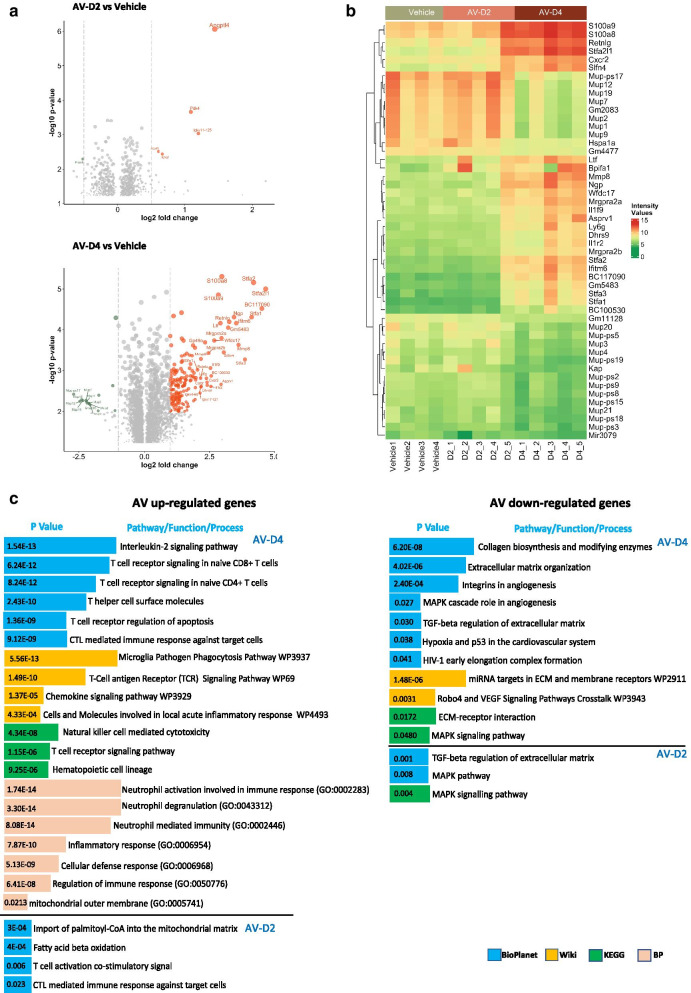
Enriched gene expression pattern of AV treatment may prevent the SARS-CoV2 infection-induced transcriptional changes** a** The volcano plot represents the variable expression status of genes upon AV-D2 and AV-D4 treatment in mice's lungs compared to the vehicle group. Red and green coloured dots represent up and down-regulated genes (FC > |2|, *P*-value < 0.05). **b** Heatmap represents the top 50 most variable genes in the AV-D4 group and their expression status in Vehicle, AV-D2, and AV-D4 group. **c **Enriched pathways and biological process in up-regulated and down-regulated genes after AV treatment compared to vehicle mice group

Lung transcriptomic analysis showed an upregulation of 1258 genes after AV-D4 treatment and 375 gens after AV-D2 treatment in naive mice. While 1133 genes in AV-D4 and 262 genes in AV-D2 were downregulated, compared to Vehicle (distilled water) treated mice (Fig. 3a, b, and Additional file 2). We observed enrichment of pathways like IL-2 signaling, T cell signaling, T cell-mediated immunity, natural killer cell-mediated cytotoxicity, Haematopoietic cell lineages in the AV-D4 up-regulated genes. Similarly, biological processes like neutrophil activation and degranulation, neutrophil-mediated immunity, immune response regulation, and cellular defense response was enriched in AV-D4 up-regulated genes (Fig. 3c). In AV-D2 up-regulated genes, pathways relevant in mitochondria, T cell signaling, cytotoxic T cell-mediated immune response are enriched (Fig. 3c). At the same time, pathways like collagen biosynthesis, extracellular matrix organization, TGF beta regulation, hypoxia, and associated inflammatory MAPK-signaling are significantly enriched in both AV-D4, and AV-D2 downregulated genes (Fig. 3c). Overall, it indicates that AV treatments favour the expression of genes important in immunity and adaptive immune response and inhibitory to genes involved in hypoxia associated angiogenesis, fibrosis, and inflammatory cascade. These findings of AV might be relevant in COVID-19 treatment, where inhibition of adaptive immune response and induction of hypoxia-inflammatory response seen in SARS-CoV2 infected patients samples [9, 10, 29–31]. Besides, increased levels of immune cells in bronchoalveolar lavage fluid (BAL) of mice treated with AV, compared to vehicle-treated mice (Additional file 1: Fig. S1b) further support our observation. Furthermore, to identify similarities and differences in AV gene expression pattern with other FDA-approved drugs and bio-actives, we mapped the transcriptomic signature of AV using Connectivity Map (CMap) database. We observed drugs like glucocorticoids, HDAC inhibitors,and NSAIDS agents (that target cyclooxygenase) have a similar gene expression patterns with AV (Additional file 1: Fig. S1c). These drugs or compounds are shown to have therapeutic potential against COVID-19 [32–35]. The possibility of such directs effects was further examined.

### In silico and in vitro* analysis* demonstrates the therapeutic potential of AV against SARS-CoV2

**Fig.4:**
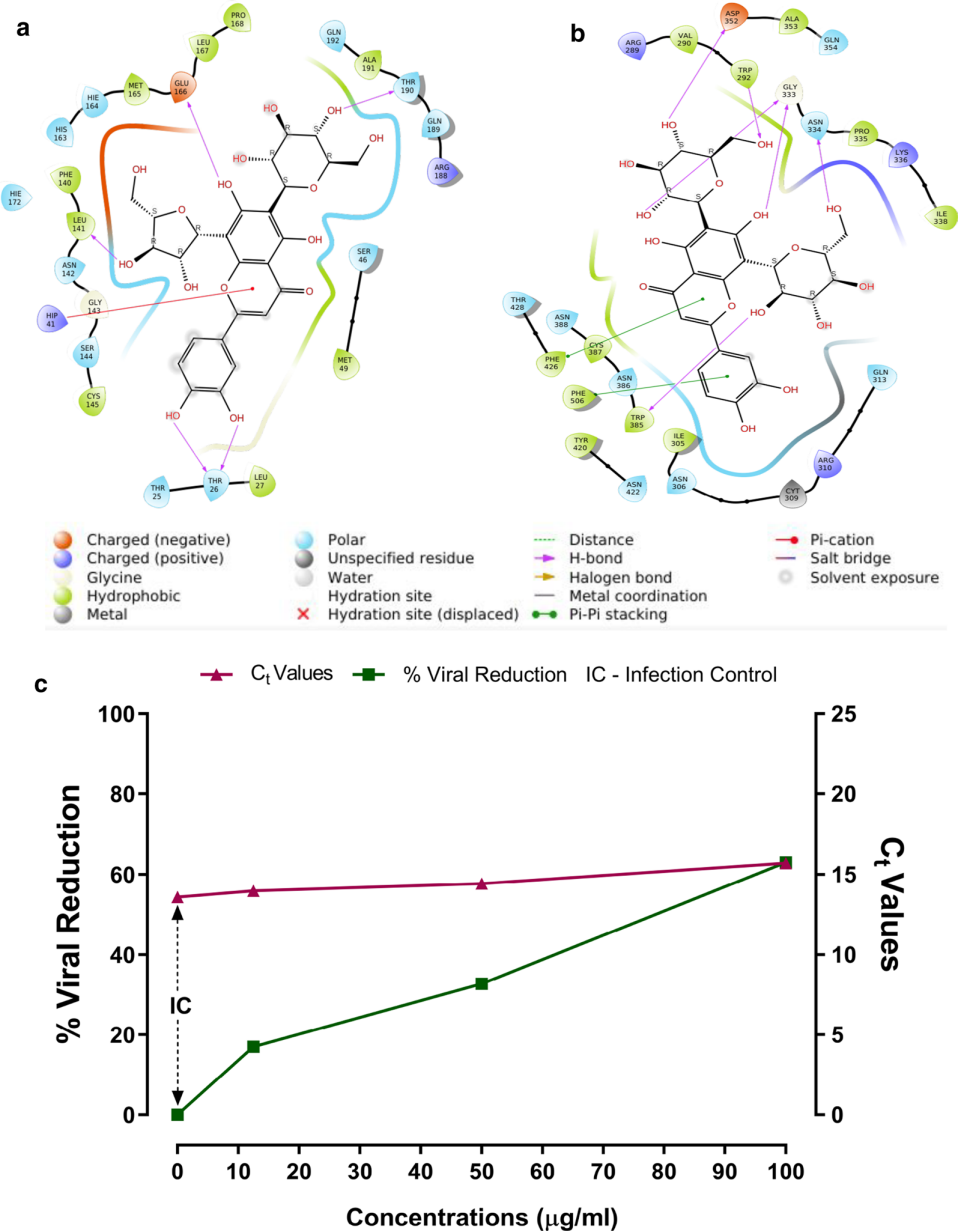
In silico and in vitro effect of AV on SARS-CoV2 targets (**a**, **b**) Molecular docking interaction of AV’s **a** Luteolin-6-C-glucoside-8-C-arabinoside with SARS-CoV2 3CLpro, and **b** Luteolin-6,8-di-C-glucoside with SARS-CoV2 NSP14 protein. **c **AV treatment inhibits the viral replication in cellular model of SARS-CoV2. Graph showing increase in Viral Reduction (%) to an increase in *C*_*t*_ Values. Y-Axis (left): Viral Reduction (%); Y’-Axis (Right): C_t_ Values; X-Axis: Concentrations of AV (µg/mL)

Chemical components present in *Adhatoda Vasica* (Additional file 1: Tables S1 and S2) were examined by molecular docking analysis with SARS-CoV2 and host proteins. Tables 1 and Additional file 1: Table S1 represent the docking results of the constituents of *Adhatoda Vasica* with key target proteins of the SARS-CoV-2 virus. The flavonoid derivatives from the extract of *Adhatoda Vasica* are found to be potential candidate against SARS-CoV-2 targets (Luteolin_6C_glucoside_8C_arabinoside has − 11.58 for 3CLpro and − 7.45 for PLpro, Luteolin-6_8-di-C-arabinoside has -9.77 for RdRp and − 13.82 for JAK2, Luteolin_6_8_di_C_glucoside has -9.42 for S-protein, − 12.08 for JAK1 and − 9.82 for ACE2, -15.25 for NSP14 and − 11.12 for TMPRSS2, Luteolin-6-C-arabinoside has − 10.57 for NSP16 and Apigenin_8_C_arabinoside has − 11.28 kcal/mol for JAK3 protein) than quinazoline alkaloids analogues such as Choline and Betaine (Table 1 and Additional file 1: Table S1). The key residues of target proteins that contribute more for binding with the compounds of AV are shown in Additional file 1: Table S2. The compound Luteolin-6-C-glucoside-8-C-arabinoside makes cation-π interaction with residue (His 41) of 3CLpro with higher affinity -11.59 kcal/mol (Fig. 4a). The π-π stacking interaction between the Luteolin-6,8-di-C-glucoside and residues (Phe 426 and Phe 506) of NSP14 protein enhances the binding affinity to -15.25 kcal/mol when compared to other compounds as shown in Fig. 4b, and other interaction plots are represented in Supporting material Additional file 1: Fig. S2, S3, and S4. In addition, AV's flavonoids are also shown to have a higher binding affinity for proteins involved in hypoxia and inflammation [16]. We also compared docking analysis of AV (Table 1) with known antivirals, JAK inhibitors and hydroxychloroquine (HCQ) against SARS-CoV2 and host target proteins (Table 2, Additional file 1: Table S3). We observed the AV’s components show a numerically higher binding affinity for SARS-CoV2 and host target proteins important for virus entry and replication than HCQ, antiviral (namely Lopinavir, Ritonavir, and Daclatasvir), and JAK inhibitors (namely Ruxolitinib, Baricitinib, Momelotinib, and Oclacitinib) compounds (Table 1 and 2). Although various JAK inhibitors have binding affinity numerically more than -9.0 kcal/mol against JAK2 protein (Table 2), the most of the flavonoid’s derivatives from extract of AV are found to have binding energy numerically more than -11.0 kcal/mol (Table 1). An anti-malarial compound HCQ (− 4.61 kcal/mol for S-protein) which obstruct the binding between ACE2 and spike protein to inhibit the invasion of SARS-CoV-2 virus [36]. Three anti-viral compounds (Lopinavir, Ritonavir and Daclatasvir) have less binding affinity when compared to flavonoids derivatives of AV with all target proteins of SARS-CoV-2 virus. In AutoDock vina, the compound Luteolin-6,8-di-C-glucoside has higher binding energy of − 9.0 kcal/mol (Additional file 1: Table S2) than other JAK inhibitors such as Ruxolitinib has − 8.2, Baricitinib has − 7.5, Memelotinib has − 8.1 and Oclacitinib has − 7.4 kcal/mol (Additional file 1: Table S3) for JAK2 protein. The trend obtained from AutoDock vina is in close agreement with previous literature [37]. Overall, results obtained from the two docking methods are found to be similar. These findings suggest that the AV extract's flavonoid derivatives are a higher binding affinity than other drugs such as anti-viral, anti-malarial, and JAK inhibitors against all target proteins of the SARS-CoV-2 virus. To confirm the *in-silico* observation, we tested the anti-SARS-CoV2 potential of AV using in vitro model of SARS-CoV2 infection. We observe a 63% viral reduction after AV treatment (100 µg/ml) in Vero cells infected with SARS-CoV2, compared to the untreated group (Fig. 4c). These results further substantiate the potential of AV in management of COVID-19.

**Table 1:** Binding affinity of compounds of *Adhatoda Vasica *with different target proteins of SARS-CoV- 2 virus in kcal/mol using Schrodinger XP Glide package

Compounds	3CLpro	PLpro	RdRp	S-pro	NSP14	NSP16	ACE2	TMPRS S2	JAK
Choline	− 2.04	− 2.304	− 3.02	− 2.403	− 1.903	− 2.721	− 6.246	− 2.314	− 1.769
Betaine	− 2.638	− 2.599	− 3.449	− 2.775	− 3.109	− 2.732	− 6.079	− 3.328	− 2.851
Vasicinol	− 4.523	− 2.542	− 2.941	− 3.187	− 5.557	− 3.848	− 3.546	− 3.591	− 7.001
Adhavasicinone	− 4.291	− 2.715	− 2.963	− 3.42	− 5.246	− 3.147	− 3.702	− 3.011	− 7.132
Linarinic_acid	− 4.547	− 3.161	− 3.486	− 3.449	− 6.048	− 4.593	− 3.539	− 4.26	− 6.181
Vasicine	− 4.027	− 2.835	− 1.961	− 2.843	− 5.313	− 3.292	− 3.026	− 3.473	− 5.973
Vasicinolone	− 4.098	− 2.787	− 3.627	− 3.207	− 5.939	− 4.303	− 3.53	− 4.371	− 7.249
5_methoxyvasicine	− 4.009	− 3.234	− 3.72	− 2.676	− 5.199	− 1.982	− 3.155	− 2.73	− 5.08
Vasicine_glycoside	− 6.387	− 3.692	− 3.803	− 4.239	− 9.359	− 5.857	− 3.964	− 6.069	− 8.374
Vasicinone	− 3.762	− 2.416	− 3.062	− 3.007	− 5.152	− 1.35	− 2.884	− 4.59	− 7.027
Luteolin_6_8_di_C_glucoside	− 9.913	− 7.451	− 9.574	− 9.429	− 15.251	− 9.894	− 9.823	− 11.124	− 12.28
Luteolin_6C_gluco side_8C_arabinoside	− 11.59	− 7.161	− 10.6	− 6.864	− 14.993	− 9.519	− 6.449	− 9.442	− 13.46
Kaempferol_3_O_rutinoside	− 8.879	− 6.889	− 7.518	− 6.606	− 12.022	− 7.604	− 5.177	− 6.888	− 11.7
Apigenin_6C_gluc oside_8C_arabinoside	− 10.57	− 6.194	− 7.23	− 5.865	− 13.67	− 8.532	− 5.762	− 6.918	− 11.67
Luteolin-6_8-di-Carabinoside	− 9.978	− 7.082	− 9.778	− 6.324	− 12.728	− 10.86	− 7.249	− 7.768	− 13.82
Luteolin_6C_gluco side	− 10.8	− 5.599	− 8.126	− 5.991	− 10.659	− 8.749	− 5.576	− 7.688	− 9.974
Apigenin-6_8-di-Carabinoside	− 11.04	− 6.062	− 8.711	− 6.011	− 12.173	− 9.899	− 4.378	− 7.286	− 10.77
Apigenin_6C_glucoside	− 8.789	− 4.01	− 7.647	− 5.016	− 9.825	− 8.23	− 4.18	− 6.826	− 8.35
Luteolin-6-Carabinoside	− 9.046	− 4.262	− 8.744	− 6.631	− 10.316	− 10.578	− 4.268	− 7.116	− 10.83
Quercetin_3_O_glucoside	− 8.119	− 6.111	− 8.233	− 7.416	− 10.757	− 7.54	− 5.078	− 7.827	− 12.49
Apigenin-8-Carabinoside	− 5.679	− 5.185	− 6.216	− 6.15	− 9.971	− 6.764	− 4.922	− 5.119	− 10.77

**Table 2:** Binding affinity of JAK inhibitors, anti-malarial and anti-viral compounds with multiple target proteins of SARS-CoV-2 virus in kcal/mol using Schrodinger XP Glide package

Compounds	3CLpro	PLpro	RdRp	S-pro	NSP14	NSP16	ACE2	TMP RSS2	JAK2	NSP4	NSP7	NSP8
JAK inhibitors												
Ruxolitinib	− 5.29	− 2.88	− 3.07	− 2.93	− 5.68	− 6.23	− 3.65	− 4	− 9.43	− 3.57	− 1.01	− 2.15
Baricitinib	− 4.62	− 2.45	− 3.41	− 3.04	− 6.98	− 5.91	− 2.1	− 3.2	-9.15	-4.06	-1.98	-2.95
Momelotinib	− 4.27	− 2.77	− 4.08	− 2.75	− 5.86	− 6.23	− 2.45	− 5	− 9.55	− 3	− 0.93	− 3.57
Oclacitinib	− 5.06	− 2.96	− 3.07	− 2.69	− 6.62	− 4.08	− 3.86	− 4.9	− 9.34	− 2.69	− 2.33	− 1.92
HCQ	− 5.97	− 4.67	− 2.64	− 4.61	− 5.98	− 4.77	− 4.67	− 4.3	− 7.25	− 4.36	− 1.43	− 2.33
Antiviral												
Lopinavir	− 5.03	− 4.99	− 3.98	− 4.32	− 9.26	− 4.26	− 2.54	− 3.9	− 6.56	− 2.36	− 0.35	− 1.8
Ritonavir	− 5.72	− 4.74	− 5.39	− 5.65	− 4.22	− 6.76	− 2.43	− 2.2	− 6.82	− 4.27	− 2.04	− 2.18
Daclatasvir	− 4.9	− 3.21	− 2.37	− 2.46	− 5.39	− 5.8	− 2.25	− 3.8	− 5.76	− 2.39	− 1.8	− 1

## Discussion

**Fig. 5:**
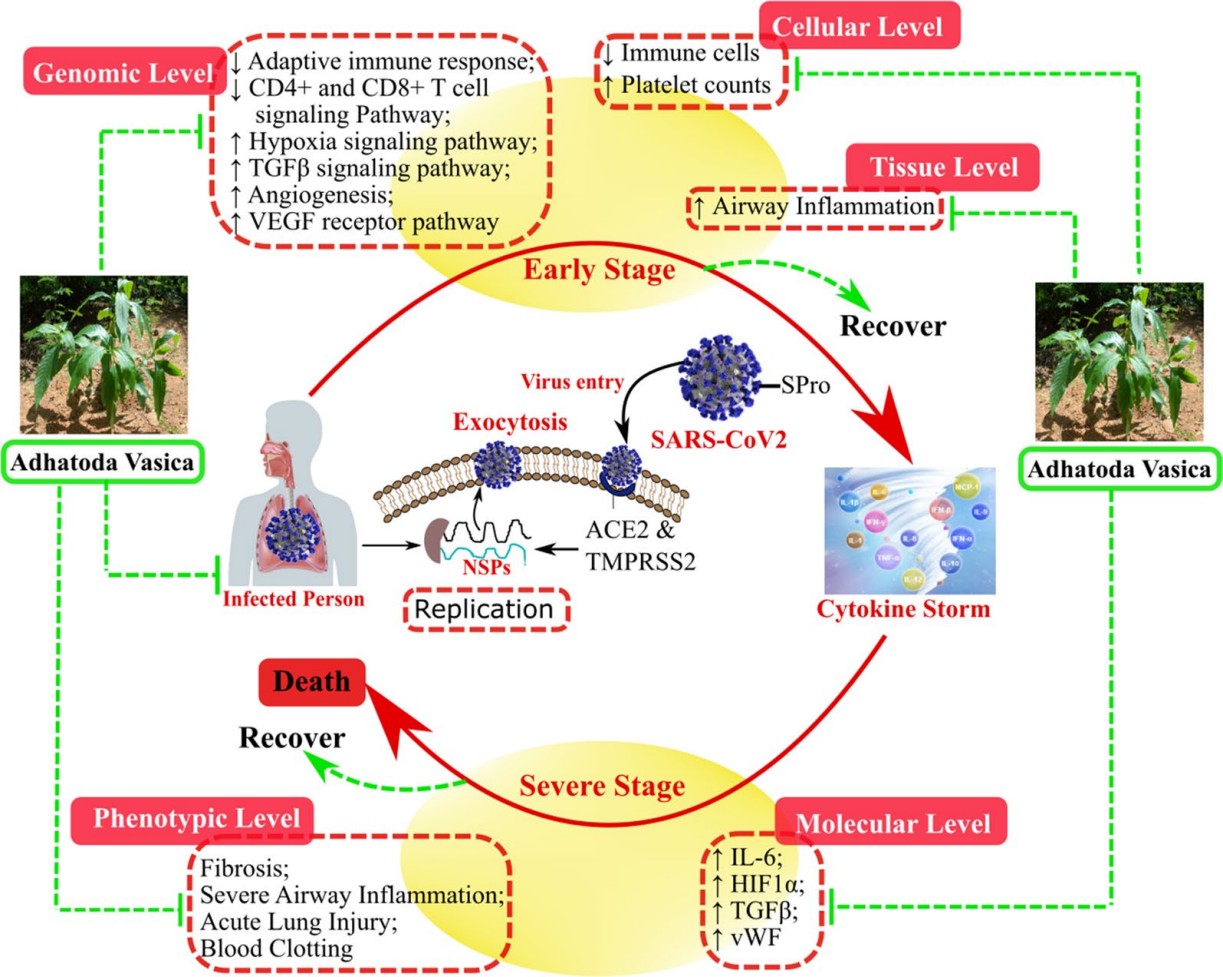
Schematic representation of multidimensional effect of AV on various markers of immunity hypoxia and inflammatory features of the lung appearing during the early as well as severe phase of COVID-19

*Adhatoda Vasica* or Vasa has been extensively used in Ayurveda for treating a wide range of inflammatory and respiratory conditions [38]. Even in modern clinical practice, it is recommended for strong bronchodilatory and antitussive effects [38, 39]. AV’s active ingredients and their derivates such as Bromhexine, and Ambroxol are effective against various respiratory ailments like asthma, COPD, and tuberculosis [38]. We have recently shown that AV alleviates the severe airway inflammation in steroid-nonresponsive asthmatic features by inhibition of HIF-1α (key transcription factor in hypoxia) via its negative regulator, PHD2. AV thereby modulates hypoxic response, which forms the basis for its diverse therapeutic effects including amelioration of mitochondrial dysfunction [16]. In this study we examined anti-hypoxic effects of AV in other hypoxia-inflammation prevalent conditions such as lung injury, fibrosis, and thrombosis. These are relevant to the current global pandemic of COVID-19, specifically progression and sequelae of SARS CoV2 infection (Fig. 5).

Our study shows that AV could reverse the pulmonary fibrosis (PF) pathological features in the bleomycin mice model (Fig. 1). HIF-1α stabilization is observed in many cell types of PF lungs and causes increase in collagen synthesis, fibrosis, TGF-β1, VEGF levels, and proliferation of fibroblasts [1, 40]. AV treatment reduces the increased expression of HIF-1α protein in bleomycin treated mice lungs and attenuates increased TGF-β1 and collagen content in mice lungs (Fig. 1a–d). These preliminary results substantiate the hypoxia modulating effect of AV in chronic lung disease conditions such as fibrosis. There were strong effects of AV treatment on inflammatory lung injury and CLP induced lung injury and mortality in mice was reduced (Fig. 1i–k). Interestingly, we observed a decrease in IFN-γ levels upon CLP in mice, which was restored after AV treatment (Fig. 1h). Our observation with the IFN-γ level in CLP mice is not in line with previous studies [41], and it is likely due to the differences in the time of measurement after CLP. We speculate that the mechanism of AV action may not be directly anti-inflammatory but rather restoration of appropriate inflammatory responses and blocking of inappropriate inflammation. This would be similar to the clinical findings that support the adjuvant IFN-γ immunotherapy concept to improve the host immune response against infection [42, 43]. AV treatment also reduced the HIF-1α induced pro-thrombotic state that may additionally be relevant to lung injury and inflammation (Fig. 2f–j).

In the course of our study, we realized that the effects of AV on phenotypic features of the lung and systemic inflammation could also prove beneficial for the present pandemic situations. In SARS-CoV2 infection, elevated hypoxia response seems to be a consequence of hyper-inflammation that contributes to disease severity [6, 7]. We relate the therapeutic relevance of AV for the above-observed effects against severe patho-phenotypes associated with the critical stage of COVID-19, characterized by severe lung inflammation, hypoxemia, angiogenesis, sepsis, and altered coagulation profile [6, 8, 11–13]. Therefore, the anti-hypoxic property of AV would be advantageous in attenuating the critical inflammatory stage of COVID-19. Our transcriptome results of AV treated mice also show down-regulation of genes related hypoxia-inflammation pathway (Fig. 3). In view of the multitude of effects of AV that may alter cellular response to infection (Fig. 5), we further determined whether there may be a direct or indirect effect of AV on SARS-CoV2 proliferation. We screened AV's chemical components against SARS-CoV2 and host target proteins and found possible interactions with both (Table 1, Additional file 1: Tables S1 and S2) [44]. The in vitro viral inhibition supports that these may be relevant (Fig. 4c). More studies are warranted to confidently determine whether there is meaningful anti-viral activity against SARS-CoV2 (Additional file 2).

## Conclusion

Treatment of *Adhatoda Vasica* extract alters the cellular hypoxic response and modulates the inflammation-thrombosis axis to reduce lung injury, thrombosis and fibrosis. Moreover, *in-silico* and in vitro analysis suggest it may be able to prevent SARS-CoV2 infection and its progression.

## Supplementary Information

**Additional file 1.** Additional tables and figures.

**Additional file 2.** Gene expression raw data (in excel format).

## Data Availability

The datasets used and/or analysed during the current study are available from the corresponding author on reasonable request. The transcriptome data from this study have been submitted to the Gene Expression Omnibus (GEO) under the accession number: GSE156759.

## Abbreviations

HIF-1α: Hypoxia inducible factor-1 α
PHD2: Prolyl hydroxylase domain 2
COVID-19: Coronavirus disease-2019
AV: Adhatoda Vasica
TGF-β1: Transforming growth factor-β1
IL-6: Interleukin-6
IL-17: Interleukin-17
IFN-γ: Interferon gamma
CLP: Cecum Ligation and Puncture
PHD2: Prolyl hydroxylase domain 2
SARS-CoV2: Severe acute respiratory coronavirus 2
PBMC: Peripheral blood mononuclear cell
BALF: Bronchoalveolar lavage fluid
ARDS: Acute respiratory distress syndrome
vWF: Von Willebrand factor
Bleo: Bleomycin treated mice
H&E: Hematoxylin and eosin
MT: Masson’s trichome
CMap: Connectivity map
nsp: Non-structural protein
3CLpro: 3C-like protease
PLpro: Papain-like protease
RdRP: RNA-dependent RNA polymerase
S-pro: Spike protein
ACE2: Angiotensin-converting enzyme 2
TMPRSS2: Transmembrane Serine Protease 2
JAK: Janus kinase
