# Supplementary material for: Adhatoda Vasica attenuates inflammatory and hypoxic responses in preclinical mouse models: potential for repurposing in COVID-19-like conditions
Source: Respir Res. 2021 Apr 6;22:99. doi: 10.1186/s12931-021-01698-9 (PMC8022127; doi:10.1186/s12931-021-01698-9)
Supplement: Supplementary file 1 — Additional file 1. Additional tables and figures. [file 12931_2021_1698_MOESM1_ESM.docx]

**Supplementary data**

**Figure S1. (A)** PHD2 levels in mice lung homogenate. Data are shown as mean ±SEM of four mice per group. **(B)** levels of total leukocyte count in the BAL fluid of the mice treated with AV. Data are shown as mean ±SEM of four mice per group (C) Alluvial plot represents gene expression similarity of AV with anti-inflammatory compounds. Left segment of plot represents compound type, middle segment represents main gene target connected between AV and listed compounds, and right segment of plot represents similarity score.

**Figure S2: Interaction of lead compounds of *Adhatoda Vasica* with different target proteins of SARS -CoV-2 virus.** (A) Interaction of Luteolin-6-C-glucoside-8-C-arabinoside with 3CLpro (B) Interaction of Luteolin-6,8-di-C-glucoside with PLpro (C) Interaction of Luteolin-6-C-glucoside-8-C-arabinoside with RdRp (D) Interaction of Luteolin-6,8-di-C-glucoside with S-protein.

**Figure S3: Interaction of lead compounds of *Adhatoda Vasica* with different target proteins of SARS -CoV-2 virus.** (A) Interaction of Vasicinone glycoside with NSP4 (B) Interaction of Kaempferol-3-O-rutinoside with NSP7 (C) Interaction of Kaempferol-3-O-rutinoside with NSP8 (D) Interaction of Luteolin-6,8-di-C-glucoside with NSP14.


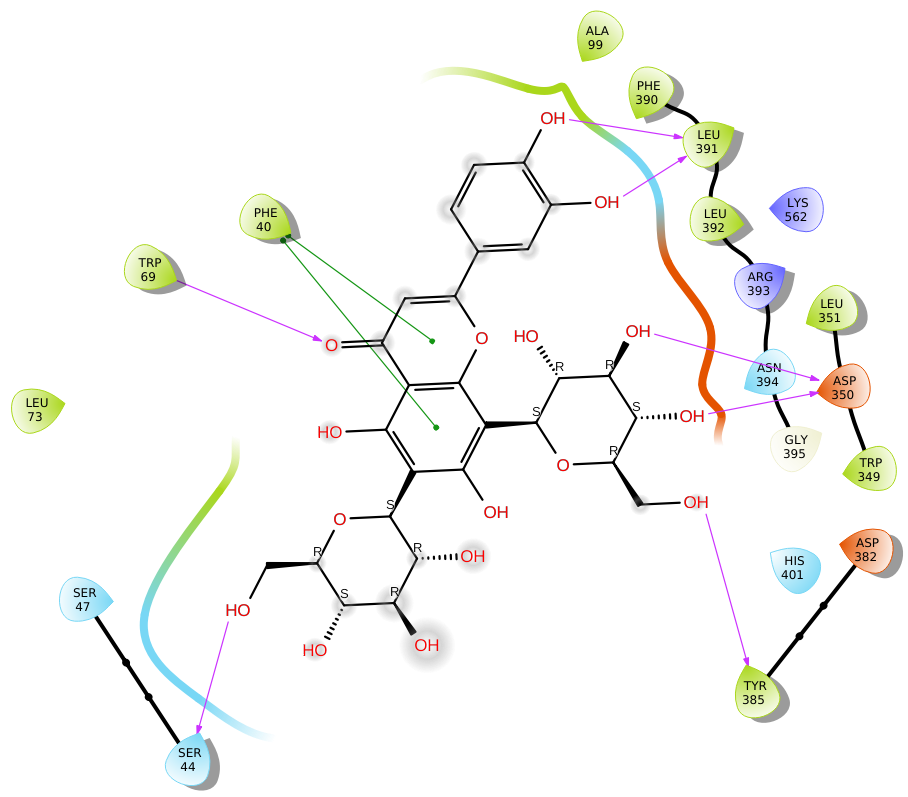


**Figure S4:** Interaction plot of Luteolin-6,8-di-C-glucoside with ACE2 receptor of human cell with binding affinity of -9.8 kcal/mol. The compound Luteolin-6,8-di-C-glucoside has 7 hydrogen bonding interactions with residues Ser44, Trp69, Asp350,Asp382 and Leu391. Notably, two π-π interactions with residue Phe40 of ACE2 protein of host cell.

**Figure S5**: **(A)** H&E stained mouse lung section. (black arrow indicates inflammation and red arrow indicates blood exudation). **(B)** H&E stained mouse lung and liver section.

**Table S1:** Binding affinity of compounds of *Adhatoda Vasica* with different target proteins of SARS-CoV-2 virus in kcal/mol using AutoDock vina

| **Compounds** | **3CLpro** | **PLpro** | **RdRp** | **S-pro** | **NSP9** | **NSP13** | **NSP14** | **NSP15** | **NSP16** | **ACE2** | **TMPRSS2** | **JAK** |
| --- | --- | --- | --- | --- | --- | --- | --- | --- | --- | --- | --- | --- |
| Choline | -3.2 | -3.3 | -3.7 | -3.1 | -2.4 | -3.2 | -3.5 | -3.1 | -3.5 | -2.7 | -3.1 | -2.9 |
| Betaine | -3.3 | -3.5 | -3.9 | -3.4 | -2.5 | -3.4 | -3.7 | -3.7 | -3.5 | -3.1 | -3.2 | -3 |
| Vasicinol | -6.3 | -5.6 | -6.5 | -5.7 | -5 | -6.4 | -7.2 | -5.9 | -6.5 | -5.2 | -6 | -6.9 |
| Adhavasicinone | -6.5 | -5.5 | -6.4 | -5.9 | -5.4 | -6.6 | -7.6 | -5.9 | -6.9 | -5.7 | -5.8 | -7.3 |
| Linarinic_acid | -6.5 | -5.5 | -6.1 | -6.1 | -5.3 | -6.8 | -7.4 | -6.4 | -6.9 | -6.6 | -6.2 | -7.2 |
| Vasicine | -6 | -5.4 | -6.2 | -5.6 | -5.3 | -6.3 | -7.7 | -6 | -6.3 | -6 | -5.7 | -6.7 |
| Vasicinolone | -6.5 | -5.8 | -6.3 | -6 | -5 | -6.8 | -7.5 | -6.5 | -6.7 | -6.4 | -6.1 | -7.3 |
| 5_methoxyvasicine | -6.4 | -5.3 | -6.7 | -5.5 | -5 | -6.6 | -6.8 | -6 | -6.9 | -6.1 | -5.7 | -6.6 |
| Vasicine_glycoside | -7.3 | -6.3 | -7.8 | -6.6 | -5.3 | -7.6 | -8.8 | -7 | -7.8 | -7.2 | -7.4 | -8.2 |
| Vasicinone | -6.2 | -5.5 | -6.5 | -6 | -5.2 | -6.4 | -7.5 | -6.3 | -6.7 | -5.9 | -6.1 | -7.3 |
| Luteolin_6_8_di_C_glucoside | -8.8 | -7 | -8.1 | -7.2 | -5.3 | -6.7 | -9.2 | -6.9 | -8.1 | -6.5 | -7.5 | -9 |
| Luteolin_6C_glucoside_8C_arabinoside | -8.2 | -7 | -7.8 | -6.8 | -4.9 | -6.5 | -9.1 | -7 | -7.8 | -6.7 | -8.1 | -8.9 |
| Kaempferol_3_O_rutinoside | -8.9 | -7.3 | -7.9 | -7.6 | -5.7 | -8.3 | -10.5 | -8.1 | -8.8 | -6.3 | -8.4 | -8.6 |
| Apigenin_6C_glucoside_8C_arabinoside | -8.3 | -6.8 | -7.8 | -6.8 | -4.6 | -6.3 | -10.3 | -6.7 | -7.5 | -6.6 | -8.1 | -8.9 |
| Luteolin-6_8-di-C-arabinoside | -7.4 | -6.6 | -8.2 | -7.1 | -5.1 | -6.4 | -9.9 | -7.4 | -8 | -6.5 | -7.6 | -8.2 |
| Luteolin_6C_glucoside | -8.6 | -6.7 | -7.5 | -6.9 | -4.9 | -7.3 | -9.9 | -7.5 | -8.5 | -7.2 | -8.3 | -8.1 |
| Apigenin-6_8-di-C-arabinoside | -7.5 | -6.5 | -7.9 | -6.8 | -5.1 | -6.1 | -10 | -7.1 | -8.1 | -6.2 | -7.6 | -8.1 |
| Apigenin_6C_glucoside | -8.3 | -6.6 | -7.5 | -6.8 | -4.8 | -7.4 | -10.1 | -7.7 | -8.4 | -7.1 | -8.4 | -8 |
| Luteolin-6-C-arabinoside | -8 | -6.9 | -7.7 | -6.4 | -4.8 | -8.4 | -9.3 | -7.7 | -8.8 | -6.7 | -8 | -7.8 |
| Quercetin_3_O_glucoside | -8.1 | -6.9 | -7.8 | -6.3 | -4.9 | -7.9 | -9.4 | -7 | -7.8 | -6.4 | -7.4 | -8.2 |
| Apigenin-8-C-arabinoside | -7.4 | -6.4 | -7.2 | -7.2 | -5.4 | -8.6 | -9.4 | -6.8 | -8.3 | -6.3 | -7 | -8.8 |

**Table S2:** Interaction of active compounds of Adhatoda vasica with important target proteins of SARS-CoV-2 virus.

| **Proteins** | **Compounds** | **Interacting Residues** | **No. of H- bonds** | **Docking Score in kcal/mol** |
| --- | --- | --- | --- | --- |
| 3CLpro | Luteolin-6-C-glucoside-8-C-arabinoside | Thr 26, Leu 141, Glu 166, Thr 190 & pi-cation (Hip 41) | 5 | -11.59 |
| PLpro | Luteolin-6,8-di-C-glucoside | Leu 162, Asp 164, Asn 267, Thr 301 | 4 | -7.45 |
| RdRp | Luteolin-6-C-glucoside-8-C-arabinoside | Asp 452, Thr 619, Lys 621, Asp 760, Asp 761 & pi-cation (Arg 553) | 8 | -10.6 |
| S-protein | Luteolin-6,8-di-C-glucoside | Arg 403, Glu 406, Gln 409, Lys 417, Tyr 453, Ser 494, Tyr 505 | 9 | -9.43 |
| NSP4 | Vasicinone glycoside | Asn 174, Asp 195, Arg 228, Asp 484 | 5 | -7.2 |
| NSP7 | Kaempferol-3-O-rutinoside | Gln 31, Glu 50, Ser 54, Ser 57, Ser 61 | 6 | -3.91 |
| NSP8 | Kaempferol-3-O-rutinoside | Ser 8, Glu 60 | 3 | -4.16 |
| NSP14 | Luteolin-6,8-di-C-glucoside | Trp 292, Gly 333, Asn 334, Asp 352, Trp 385 & pi-pi stacking (Phe 426, Phe 506) | 6 | -15.25 |
| NSP16 | Luteolin-6,8-di-C-arabinoside | Asp 6928, Tyr 6930, Asp 6873, Asp 6897, Leu 6898 | 7 | -10.86 |
| TMPRSS2 | Luteolin-6,8-di-C-glucoside | Val 280, Hie 296, Asn 303, Glu 388, Gly 439 | 8 | -11.12 |
| JAK2 | Luteolin-6,8-di-C-arabinoside | Glu 930, Leu 932, Asp 939, Arg 980, Asn 981 | 7 | -13.82 |

**Table S3:** Binding affinity of JAK inhibitors, anti-malarial and anti-viral compounds with multiple target proteins of SARS-CoV-2 virus in kcal/mol using AutoDock vina.

| **Compounds** | **3CLpro** | **PLpro** | **RdRp** | **S-pr0** | **NSP14** | **NSP16** | **ACE2** | **TMPRSS2** | **JAK2** | **NSP4** | **NSP7** | **NSP8** |
| --- | --- | --- | --- | --- | --- | --- | --- | --- | --- | --- | --- | --- |
| **JAK inhibitors** |  |  |  |  |  |  |  |  |  |  |  |  |
| Ruxolitinib | -7.3 | -6 | -6.6 | -6.2 | -8.4 | -8.1 | -6.4 | -6.6 | -8.2 | -7.6 | -4.9 | -5.1 |
| Baricitinib | -7.7 | -6.5 | -6.9 | -6.4 | -8.1 | -8 | -6.1 | -7 | -7.5 | -7.2 | -4.8 | -5.1 |
| Momelotinib | -8 | -6.6 | -7.1 | -7.3 | -9.5 | -8.9 | -6.4 | -7.9 | -8.1 | -7.7 | -5.8 | -6 |
| Oclacitinib | -7 | -6.4 | -6.5 | -5.6 | -7.6 | -7.3 | -5.5 | -6.3 | -7.4 | -6.3 | -4.9 | -4.6 |
| **HCQ** | -5.9 | -5.6 | -5.9 | -5.4 | -7.5 | -6.6 | -6.3 | -5.8 | -6.9 | -6.3 | -4.5 | -4.4 |
| **Antiviral** |  |  |  |  |  |  |  |  |  |  |  |  |
| Lopinavir | -8.1 | -6.9 | -7.6 | -7 | -10.1 | -8.4 | -6.1 | -7.6 | -8.5 | -7.6 | -4.9 | -6.1 |
| Ritonavir | -7.7 | -6.1 | -7.3 | -6.1 | -9.7 | -8.3 | -6.4 | -7.4 | -8.2 | -7.5 | -5 | -5.6 |
| Daclatasvir | -7.4 | -8.4 | -8 | -7.7 | -9 | -7.7 | -6.1 | -8.7 | -8.2 | -6.7 | -4.4 | -6 |
